# The Parameter Calibration of Social Force Model for Pedestrian Flow Simulation Based on YOLOv5

**DOI:** 10.3390/s24155011

**Published:** 2024-08-02

**Authors:** Tianle Li, Bingbing Xu, Weike Lu, Zidan Chen, Sizheng Zhang, Fanjun Xia

**Affiliations:** School of Rail Transportation, Soochow University, Suzhou 215000, China; tl_li@foxmail.com (T.L.); xbbxbb0106@163.com (B.X.); 2147401022@stu.suda.edu.cn (Z.C.); zhangsz0128@163.com (S.Z.); xiafanjun1208@163.com (F.X.)

**Keywords:** social force model, YOLOv5, parameter calibration, optimization

## Abstract

With the increasing importance of subways in urban public transportation systems, pedestrian flow simulation for supporting station management and risk analysis becomes more necessary. There is a need to calibrate the simulation model parameters with real-world pedestrian flow data to achieve a simulation closer to the real situation. This study presents a calibration approach based on YOLOv5 for calibrating the simulation model parameters in the social force model inserted in Anylogic. This study compared the simulation results after model calibration with real data. The results show that (1) the parameters calibrated in this paper can reproduce the characteristics of pedestrian flow in the station; (2) the calibration model not only decreases global errors but also overcomes the common phenomenon of large differences between simulation and reality.

## 1. Introduction

Traffic volume has increased dramatically. The subway has an important role in modern transportation systems [1]. The subway has the characteristics of large transport capacity, fast speed, high punctuality rate, reliable security, and excellent comfort. It attracts a large volume of pedestrian flow. Pedestrian flow, especially in the morning and evening peak hours, increases the requirements for the operational capacity of urban rail transit. Therefore, grasping the rules of pedestrian flow helps cities to make better plans.

Many remarkable models that simulate the complex behavior of pedestrians have been proposed to analyze movement processes in different situations. Pedestrian dynamics provides a theoretical basis for analyzing passenger motion in different situations. Pedestrian dynamics modeling consists mainly of macroscopic and microscopic models. Kaldawi [2] investigated the mathematical theory of Hughes’ model. SPH [3], a meshless method, was proposed by Yuan et al. In this method, the population is represented by a set of particles that have material properties and move according to macroscopic laws. The macroscopic model [4] also includes the DTA model improved in recent years by Aghamohammadi and Laval. These models usually view crowd motion ideally as the flow of a liquid or gas, most commonly fluid or gas dynamics. However, for practical situations, this view of a uniform distribution of liquid or gas is erroneous. The reason for this is that pedestrians display interactive behaviors and behavioral habits that cannot be represented by macro models. The microscopic model can fully consider the heterogeneity of pedestrians and the interaction between pedestrians in order to overcome the shortcomings of the macroscopic model, which is more suitable for studying the behavior of passengers in the station.

Macroscopic models [5] are not suitable for predicting pedestrian movement in pedestrian areas or buildings, unlike microscopic models, which are more widely used and consider design details. Vermuyten et al. [6] provided a program for the calibration and implementation of the model. Babojelić and Novacko [7] emphasize that pedestrian behavior has a significant impact on traffic safety and traffic flow at both micro and macro levels. The magnetic force model [5] defines the pedestrian and destination as the cathode and anode of a magnetic field, respectively. In the model, the attraction and repulsion forces are calculated by Coulomb’s law. The queuing theory model [8] is more suitable for simulating the bottleneck effect and can intuitively visualize the simulation effect. But when the crowds are overcrowded, the simulation results are often less accurate. In the cellular automata model [7], pedestrians are recognized as individuals, and the moving areas are divided into cells, which results in the simulation accuracy being impacted by the cell scale.

The social force model has also received improvements [9,10,11]. Li et al. [12] proposed an improved social force model where pedestrians consider avoiding potential conflicts in advance during the walking process, making pedestrian trajectories smoother and more realistic. An et al. [13] also considered the different viewpoint sensitivities of pedestrians to other obstacles and humans based on the original model. They proved the efficiency of the model in the complex scenario of passengers alighting and boarding the metro. In order to solve the problem of the slow simulation speed of the social force model in complex scenarios, Yang et al. [14] modified the forces between the pedestrians and obstacles in the model. By finding the optimal number of obstacle iterations, this method increased the speed of the model simulation.

In recent years, most of the parameter calibration studies of the social force model have been aimed at better modeling pedestrian evacuation (Table 1). For example, Sticco et al. [15] and Kanté et al. [16] both calibrated the parameters of panicked pedestrians in emergency evacuations. This means that the results they calibrated can only be applied to limited data sets. And the algorithms they use are differential evolution and genetic algorithms, which result in slow convergence velocity and a long solution process. In the studies [17,18] on the calibration of pedestrian parameters in metro stations, the parameters were obtained by formula calculation. Most existing calibration models are conducted based on full-self-investigated data. They cannot capture pedestrian dynamics. These models focus on improving simulation accuracy on average pedestrian flow variables (e.g., speed, flow, and density) but ignore the spatial distribution diversity of pedestrian flow in stations. In this study, we employed YOLOv5 [19] to map the spatial distribution of pedestrian flow in subway stations. Then, we established an image matrix to describe the flow characteristics. This reflects the spatial distribution diversity of pedestrian flow. Also, this study built a calibration model solved by the improved Newton downhill method to reduce the calibration cost.

In this study, the parameter ranges were first derived from the available research. Next, this method required field research scenarios to obtain the pedestrian data and actual video. The actual video was imported into YOLOv5 for image recognition, and the final recognition results for each video were presented in the form of a density map. Then, pedestrian data were input, along with an initial set of parameters, into the simulation software Anylogic (Version 8.7.0), obtaining a density map. With the help of the OpenCV library, two density maps were transformed into two image matrices. We determined the convergence condition by calculating the size of the error value of the two matrices. The parameters were iterated continuously by the Newton downhill method until the convergence criterion was reached. 

Finally, a set of model parameters more realistic with the actual conditions of pedestrians were obtained and verified for accuracy. This study has a specific guiding significance for the operation management of the subway station and enhances the sustainability of urban transportation.

## 2. Social Force Model

**Figure 1:**
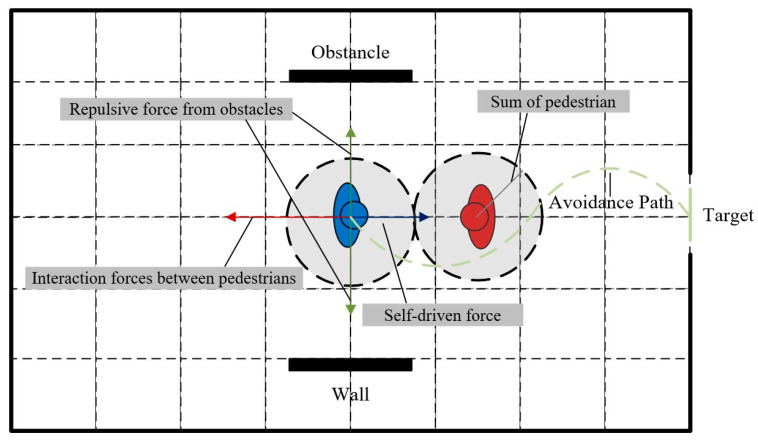
The diagram of the social force model.

In 1951, Helbing constructed the social force pedestrian flow model (Figure 1) on the basis of fluid dynamics. Now, the model is widely accepted by research scholars. The model points out that in the process of going to the target, pedestrians are subjected to various forces, including their own driving force, the interaction force between pedestrians, the interaction force between pedestrians and surrounding obstacles, attractive force, etc. Through the analysis of forces in physics, the model can explain the behaviors of pedestrians of different choices in the process of movement. The specific formula is given as

(1) $$\vec{f_{i}}\left(t\right)=\vec{f_{i}^{o}}\left(t\right)+\vec{f_{ij}}\left(t\right)+\vec{f_{iw}}\left(t\right)$$

(2) $$\vec{f_{ij}}\left(t\right)=\vec{f_{ij}^{soc}}\left(t\right)+\vec{f_{ij}^{ph}}\left(t\right)$$

(3) $$\vec{f_{iw}}\left(t\right)=\vec{f_{iw}^{soc}}\left(t\right)+\vec{f_{iw}^{ph}}\left(t\right)$$

where $\vec{f_{i}}\left(t\right)$ donates the sum of the forces acting on pedestrian i at moment t; $\vec{f_{i}^{o}}\left(t\right)$ donates self-driven force on pedestrian i at moment t;$\vec{{\;f}_{ij}}\left(t\right)$ donates force on pedestrian i at moment t from other pedestrians j in the vicinity; $\vec{f_{iw}}\left(t\right)$ donates force on pedestrian i at moment t from obstacle b; and $\vec{f_{ij}^{soc}}\left(t\right)$, $\vec{f_{ij}^{ph}}\left(t\right)$, $\vec{f_{iw}^{soc}}\left(t\right)$, and $\vec{f_{iw}^{ph}}\left(t\right)$ reflect the psychosocial force and physical force of pedestrian i by other pedestrians j in the neighborhood or obstacle w, respectively, at moment t.

(4) $$\vec{f_{i}^{o}}\left(t\right)=m_{i}\frac{v_{i}^{o}\vec{e_{i}^{o}}\left(t\right)-\vec{v_{i}}\left(t\right)}{\tau_{i}}$$

(5) $$\;\vec{f_{ij}}\left(t\right)=A_{i}exp\left[\frac{r_{ij}-d_{ij}}{B_{i}}\right]\vec{n_{ij}}+K\theta \left(r_{ij}-d_{ij}\right)\vec{n_{ij}}+K\theta \left(r_{ij}-d_{ij}\right)∆v_{ji}^{t}\vec{t_{ij}}$$

(6) $$r_{ij}=\left(r_{i}+r_{j}\right)$$

(7) $$\vec{n_{ij}}=\frac{\left[r_{i}\left(t\right)-r_{j}\left(t\right)\right]}{d_{ij}\left(t\right)}$$

(8) $$\vec{f_{iw}}\left(t\right)=A_{w}exp\left[\frac{r_{i}-d_{iw}}{B_{w}}\right]\vec{n_{iw}}+K\theta \left(r_{i}-d_{iw}\right)\vec{n_{iw}}+K\theta \left(r_{i}-d_{iw}\right)∆v_{wi}^{t}\vec{t_{iw}}$$

where $v_{i}^{o}$ and $\tau_{i}$ are the desired speed and relaxation time of pedestrian i respectively; $A_{i}$ and $A_{w}$ reflect the interaction intensity of other pedestrians and obstacles, respectively; $B_{i}$ and $B_{w}$ correspond to the interaction range of other pedestrians and obstacles; $r_{i}$, $r_{j}$ denote the radii of pedestrian i and pedestrian j; $d_{ij}$ is the center distance between two pedestrians i and j; $d_{iw}$ is the distance between obstacle w and pedestrian i; $\vec{n_{ij}}$ is the direction of repulsive force of pedestrian j on pedestrian i; $\vec{n_{iw}}$ is the direction of continuity between obstacle w and pedestrian i; $K\theta (r_{ij}-d_{ij})\vec{n_{ij}}$ and $K\theta (r_{ij}-d_{ij})∆v_{ji}^{t}\vec{t_{ij}}$ are the physical repulsive and friction forces between pedestrian i and j; $K\theta (r_{iw}-d_{iw})\vec{n_{iw}}$ and $K\theta (r_{iw}-d_{iw})∆v_{wi}^{t}\vec{t_{iw}}$ are the physical forces to the pedestrian i, which are exerted by obstacle w when $\left(r_{i}-d_{iw}\right)$ is larger than zero. In this study, $v_{i}^{o}$, $\tau_{i}$, $A_{i}$, and $B_{i}$ are selected as parameters in vector ***P*** and are calibrated through the proposed optimization model. Related studies on parameter calibration of the social force model [17,20] were compiled and summarized. Finally, a set of initial parameter value ranges are integrated in Table 2.

## 3. Calibration Model

**Figure 2:**
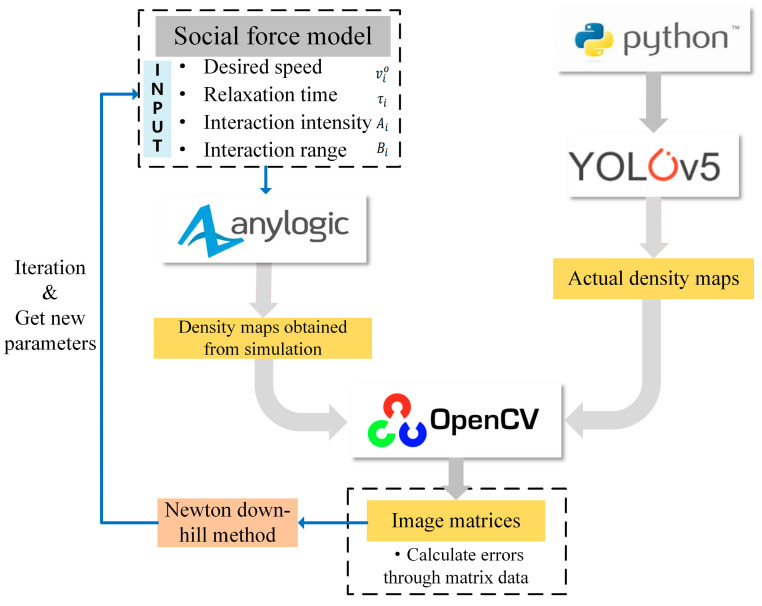
The overall framework of the calibration model.

The overall framework of the calibration model is depicted in Figure 2. Anylogic provides a feasible and effective tool for the simulation experiment of pedestrian movement at the subway station. The movement of pedestrians is based on the social force model. For the parameters to be calibrated in the vector ***P***, Ko et al. [21] considered the parameters at which the probability function took its maximum value as the calibration parameters using maximum likelihood estimation. Sticco et al. [15] applied differential evolution for continuous iteration to obtain optimal values. However, the above methods are computationally intensive and the solution process is complicated. Therefore, a calibration method that is low in workload and simple is necessary.

### 3.1. Evaluation Map

In this study, all four parameters required to be calibrated reflect the spatial characteristics of pedestrian motion. Therefore, the parameters in the calibration model must accurately represent the spatial variability of pedestrians. Considering the aforementioned conditions and accessibility, we selected the pedestrian flow density map as the model parameter. The density map not only reflects the passenger’s travel state but can also be generated by YOLOv5. YOLOv5 [22] performs effectively across diverse scenarios and can adapt to complex environments. Consequently, generating density maps through YOLOv5 has great applicability. YOLOv5’s models take up less storage space, enabling fast operations and adaptability on resource-limited equipment. Gai et al. [23] and Li et al. [24] applied YOLOv5 to pedestrian target tracking, ultimately enhancing recognition accuracy. However, there is no quantitative standard for straightforwardly comparing the density maps. Therefore, this model introduces an image matrix for quantifying density maps.

**Figure 3:**
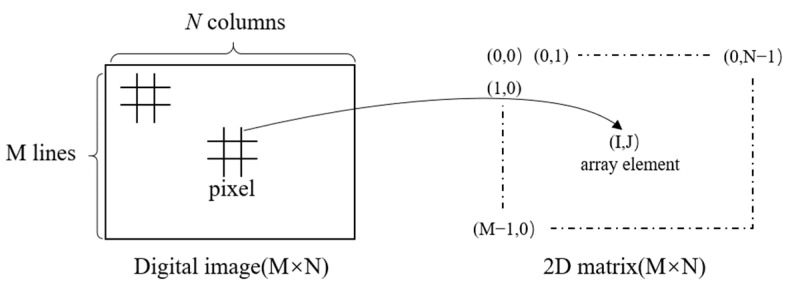
The diagram of the image matrix.

For any image, it can be represented by a set of data in various color modes. Thus, an image can be represented by a matrix containing extensive data. Python’s OpenCV library [25] facilitates converting an image to a 3D matrix representation (Figure 3). By comparing corresponding data positions in matrices of two same-sized images, differences between them can be determined.

### 3.2. Calibration Model

Initially, this model defines the vector [$v_{i}^{o}$, $\tau_{i}$, $A_{i}$, $B_{i}$], denoted as ***P***, comprising the four parameters for calibration. The primary objective of the model is to iteratively find the appropriate parameter vector ***P*** during continuous simulation, aiming to minimize the error between the density map derived from Anylogic simulation and the actual density map. In this paper, the set of data groups is denoted as *K*. Here, $x_{ij,k}$ and $x_{ij,k}^{real}$ represent the values at position i,j in the group k of simulated and real matrices, respectively, while $F_{k}$ signifies the error of group k.

(9) $$\min Z\left(t\right)=\sum_{k\in K}\sum_{i=1}^{m}\sum_{j=1}^{n}({{x_{ij,k}-x}_{ij,k}^{real})}^{2}$$

(10) $$s.t.\;\left|{F_{k}-F}_{k}^{real}\right|\leq \varepsilon \;\;\;\;\;\forall k\in K$$

(11) $$\;F_{k}=\frac{1}{m\times n}\sum_{i=1}^{m}\sum_{j=1}^{n}\frac{{x_{ij,k}-x}_{ij,k}^{real}}{x_{ij,k}^{real}}\;\;\;\;\forall k\in K$$

(12) $$\;\;x_{ij,k}=Anylogic\left(P,K\right)\;\;\;\;\forall k\in K\;$$

(13) $${\;\;\;\;\;\;\;\;\;\;\;\;\;\;\;\;\;\;\;\;\;\;\;\;\;\;\;\;\;\;\;\;\;\;\;\;\;\;\;\;\;\;\;\;\;\;\;\;\;\;\;\;P}_{min}\leq P\leq P_{max}\;\forall P\in R^{n}$$

Equation (9) is the objective function of the model, which indicates that the sum of the squares of the errors of all the simulated data and the real data is minimized. Equation (10) ensures that the relative error of the density map within any group remains below ε. Equation (11) details the calculation method for $F_{k}$. Equation (12) indicates that $x_{ij,k}$ is simulated by Anylogic, and Anylogic denotes the simulation function of the simulation software Anylogic, as well as the basic input parameters of Anylogic simulation, including the parameter vector ***P*** and the groups *K*. Equation (13) restricts the values of the parameters and controls them in a reasonable range of values. For models that require multiple iterations to be solved, most use genetic algorithms for solving. But in this paper, in order to simplify the difficulty of solving, this model resorts to the Newton downhill method [26] for the model solution, and the iterative formula is

(14) $$\alpha^{i+1}=\alpha^{i}-\left|\frac{F_{i}-F_{0}}{\alpha^{i}-\alpha^{0}}\right|\cdot ∆\propto \cdot \omega_{\alpha }\;\;\;\alpha \in P,if\;\left(F_{i}-F_{0}\right)\geq 0$$

(15) $$\alpha^{i+1}=\alpha^{i}+\left|\frac{F_{i}-F_{0}}{\alpha^{i}-\alpha^{0}}\right|\cdot ∆\propto \cdot \omega_{\alpha }\;\;\;\;\alpha \in P,\;if\;\left(F_{i}-F_{0}\right)\leq 0$$

where $\alpha^{i+1}$ is the parameter α after i iterations; ∆∝ reflects the step size, and in this paper, we fix it to 1; $\omega_{i}$ denotes the weight of the parameter α, and this method determines the weights based on the magnitude of changes in these parameters, as shown in Table 3 below; and $F_{i}$ denotes the error after i iterations. At the same time, each time it is tested if the newly generated solution satisfies the constraint Equation (13).

**Figure 4:**
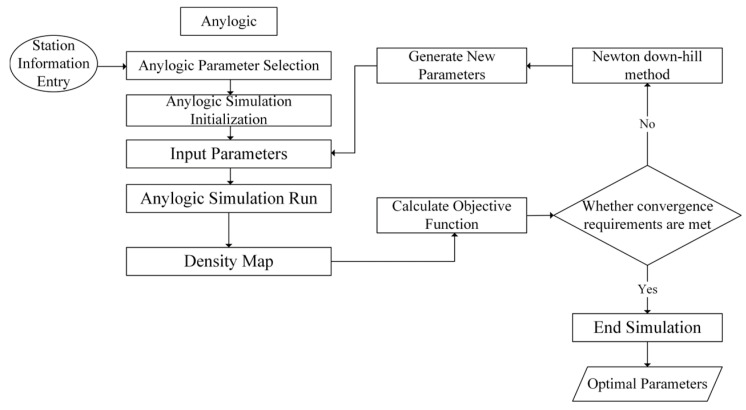
Algorithm flow chart.

The solution method (Figure 4) is as follows:

**Step 0**: Anylogic simulation initialization, such as building the subway station model, setting the simulation time, etc. We list the iteration-stopping conditions: if the change in the objective function is less than 5% or if the maximum number of iterations is reached, the iteration is stopped.

**Step 1**: Input parameters into Anylogic.

**Step 2**: Anylogic starts the simulation, obtains a density map, and substitutes the simulated results into the objective function. Check if the convergence requirement is met; if yes, skip to step 4. If no, start step 3.

**Step 3**: Adjust the parameters through the Newton downhill method to produce a new parameter matrix; go back to step 1.

**Step 4**: Stop the iteration and end the simulation.

## 4. Case Study

**Figure 5:**
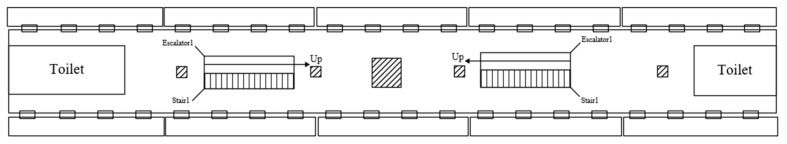
Layout of platform floor of Suzhou Pinglonglu East Station.

In order to verify the model in this paper, the platform of Pinglonglu East Station in Suzhou City was selected for parameter calibration. Figure 5 displays the schematic diagram of the station. Additionally, this study investigated the parameters associated with facilities, equipment, and pedestrians in the station, as detailed in Table 4 and Table 5.

**Figure 6:**
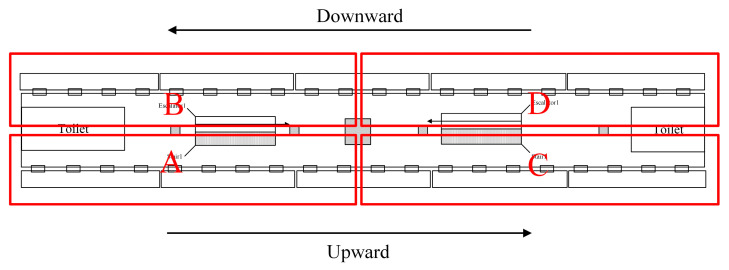
Four areas of the platform floor.

There are large public venues, including The First Affiliated Hospital of Soochow University, Wanda Plaza, Suzhou City Life Plaza, and Sunny Club near the station. This substantial pedestrian flow expands the sample size, thereby enhancing the precision of parameter calibration. The station measures 83.75 m in length and 9.75 m in width. There are only two escalators and two stairs on the platform, of which both escalators are upward. Passengers access the platform solely via two positions: Stair 1 and Stair 2. Moreover, 20 waiting areas are evenly dispersed in front of the platform screen door. Integrating the platform’s spatial layout, the experiment divides the scene into four regions labeled A-D, illustrated in Figure 6. The four regions are differentiated by the stairs and escalators numbered 1 or 2, along with their ascending or descending directions, detailed in Table 6. This partitioning ensures precise density measurements due to an enhanced recognition screen resolution and validates the applicability of calibration parameters across all zones.

### 4.1. Data Collection

The videos of pedestrians entering the platform from different escalators/stairs during the peak period (17:00–18:00) were collected through field investigation. A total of 12 groups of data were collected, and the collection time of each group was 5 min.

### 4.2. Data Processing

**Figure 7:**
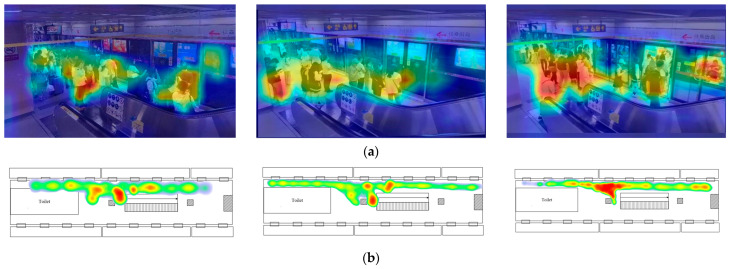
Initial actual density maps generated by YOLOv5: (**a**) Generated by YOLOv5; (**b**) Converted to 2D plane view.

Pedestrian target detection was conducted using YOLOv5 on each set of captured videos. Each group of videos subsequently produced the actual passenger density map, as shown in Figure 7a. This gave the initial actual density map, excluding the identified content outside the target area and converting it to the same form as the density map obtained from the simulation, as illustrated in Figure 7b. The colors in the density map indicate the magnitude of the density, which decreases from red to yellow, green, and blue.

### 4.3. Simulation and Results

**Figure 8:**
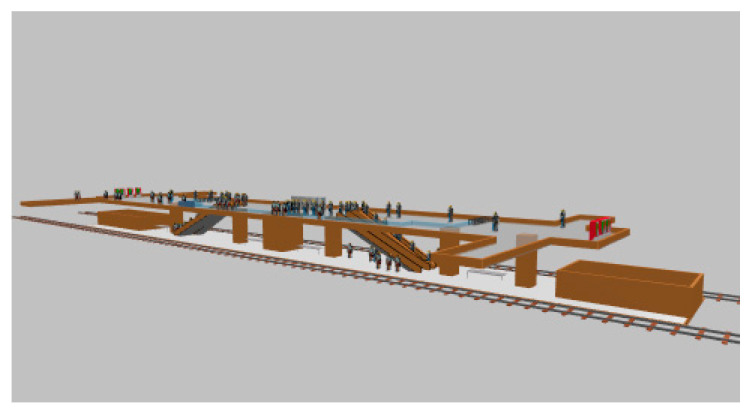
Anylogic simulation interface.

**Figure 9:**
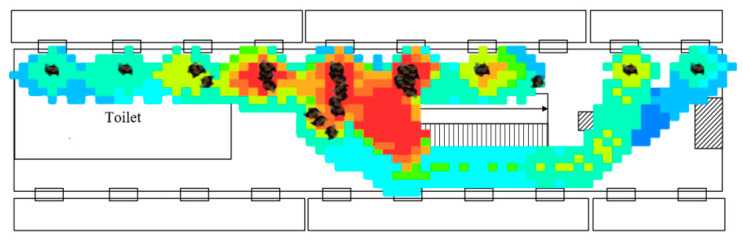
Pedestrian density map.

According to our survey, we found that area B has a higher traffic volume. Therefore, this model calibrated the parameters using area B as a baseline. The first time, the maximum and minimum values of the parameters described in the previous section were inputted in Anylogic’s social force model, and simulation was started with the simulation interface, as shown in Figure 8. After the simulation time, two density maps were obtained in Anylogic, and Figure 9 was one of them.

This model resized the density map obtained by YOLOv5 to match the dimensions of the two density maps obtained by Anylogic. Subsequently, their image matrices were acquired using OpenCV, and errors between the simulated density maps and the actual density map were calculated.

**Figure 10:**
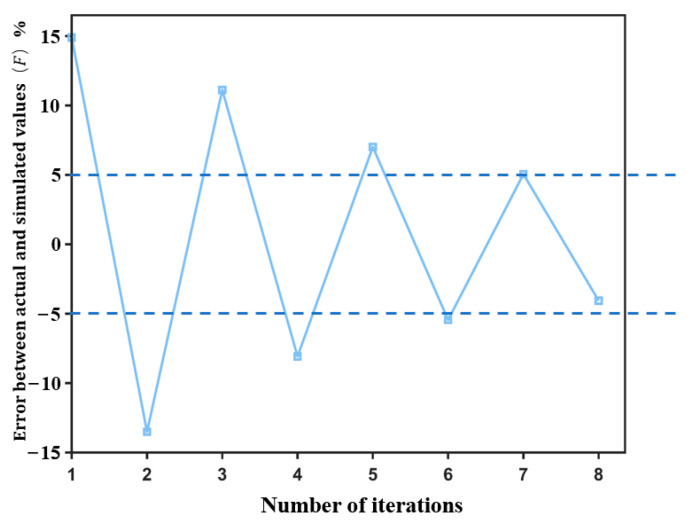
Error convergence curve.

Using these two errors, this model can start iteratively calculating using the Newton downhill method. This model continuously calibrated and optimized the parameters until the error of the image matrix converged, and the iterative process is shown in Figure 10. In the curve, the vertical axis denotes the error corresponding to each iteration, calculated by Equation (11), while the horizontal axis represents the iteration count.

The analysis of the error convergence curve reveals that the error fell below 5% after eight iterations, meeting the stopping criterion for convergence. The calibrated parameters obtained are detailed in Table 7.

**Figure 11:**
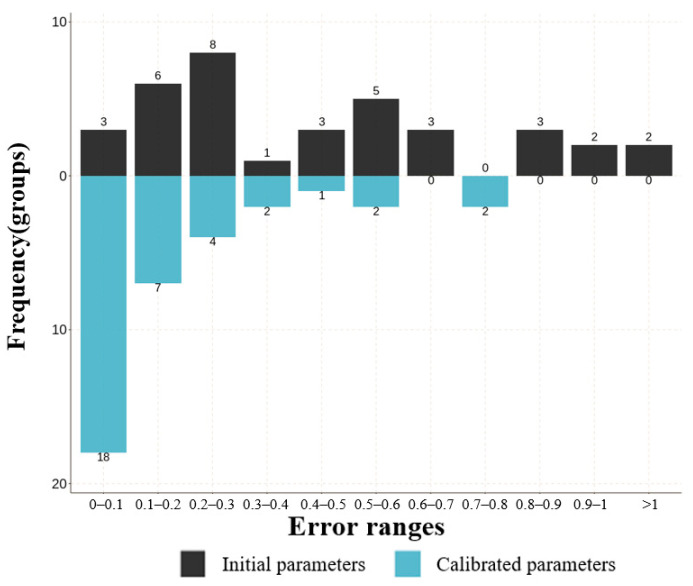
The error frequency distribution at simulation initiation and after parameter calibration in mid-demand.

To assess the accuracy of the calibration parameters, the errors in other regions were quantified before and after calibration. A total of 36 data groups (3 areas, 12 groups of data per area) were analyzed, as depicted in Figure 11. In the initial case of simulation (prior to parameter calibration), the relative error to the measured value exceeded 50% in 17 data cases, including 4 cases with errors exceeding 100%. In particular, there were four pieces of data with more than 100 percent error. Following parameter calibration, 18 data points were within 10% of the measured values, with only 4 data points exceeding a 50% error, indicating that the parameters are closer to the actual situation after the parameter calibration.

**Figure 12:**
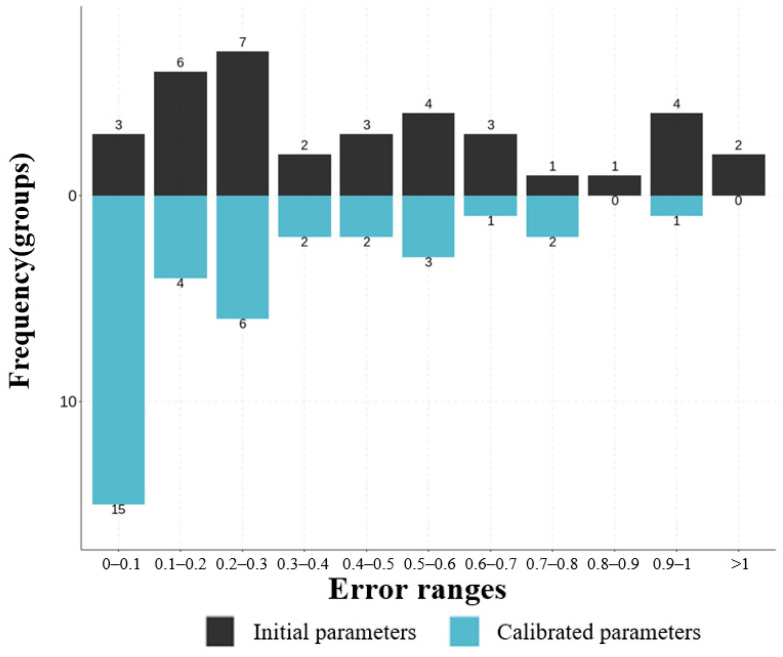
The error frequency distribution at simulation initiation and after parameter calibration in low demand.

**Figure 13:**
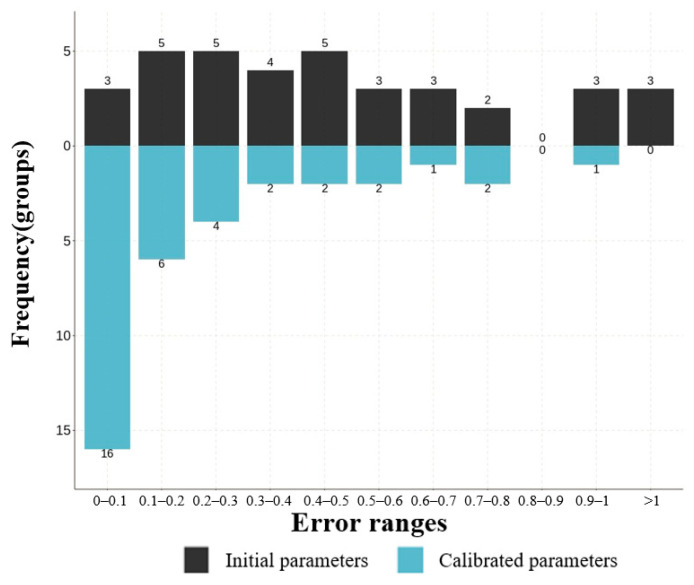
The error frequency distribution at simulation initiation and after parameter calibration in high demand.

In addition, the extreme situations were validated. By adjusting various passenger flow inputs, errors for both low- and high-demand scenarios were obtained, as seen in Figure 12 and Figure 13. The same conclusion was drawn as with the medium-demand case. Prior to parameter calibration, more than half of the data exhibited errors exceeding 50%, including cases with errors surpassing 100%. These results reaffirm the reliability and applicability of the model.

**Figure 14:**
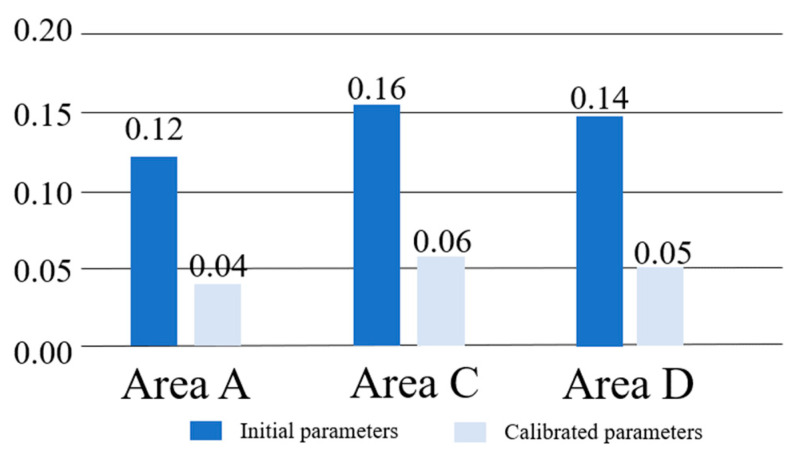
Comparison of errors in each region at simulation initiation and after parameter calibration.

Simultaneously, errors were tallied in each area, as referenced in Figure 14, revealing a notable reduction in errors in areas A, C, and D following parameter calibration compared to before calibration. Furthermore, despite fewer passengers in area D relative to other areas, the error remains approximately 5% post-calibration. An analysis of all results demonstrated that the parametric model and methodology employed herein mitigate overall error while also ensuring individual areas closely align with actual conditions.

## 5. Conclusions

In this study, a model for calibrating the parameters of a social force model based on YOLOv5 is introduced. The model is solved using Newton’s downhill method, which reduces computational complexity. It features innovative design and offers higher practical applicability, aligning closely with real-world scenarios.

Furthermore, the approach is applied based on pedestrian density maps extracted from the videos in Suzhou Pinglonglu East Station. Validation of the model is conducted at the macroscopic level using the Anylogic multi-agent simulation platform. The results demonstrate that the parameters calibrated in this method can accurately replicate pedestrian flow characteristics in the metro station. Compared with the initial parameters obtained from related works, the results are more compatible with the real data collected in this study. The calibration model used in this study actually falls under the category of multi-constraint calibration. This framework allows for the inclusion of additional calibration indices, enhancing Anylogic’s ability to finely and accurately characterize actual pedestrian flow conditions. From a practical application perspective, the method proposed in this study offers high migratability and practicality. The model itself and its algorithm for solving the objective function are straightforward and not overly complex. Given its high expandability, this study sets the stage for discussing more efficient parameter-calibration methods.

## Figures and Tables

**Table 1:** Comparison of related studies.

Publication	Applicable Scenarios	Solution Method	Pedestrian Dynamics	Pedestrian Spatial Distribution	Convenience and Flexibility of Collecting Field Data
Sticco et al. [15]	High stress	Differential Evolution Algorithm			
Kanté et al. [16]	Bidirectional corridor		Yes		
Tang and Jia [17]	Specific traffic scenarios	Least square estimation	Yes		
Rudloff et al. [18]	Transportation hubs	Least square estimation			
This paper	Full scenario	Newton downhill method	Yes	Yes	Yes

**Table 2:** Parameters of social force model integrated from different research results.

Parameter	Value	Unit
$v_{i}^{o}$	1.1–1.5	m/s
$\tau_{i}$	0.4–0.6	s
$A_{i}$	9–12	ms^−2^
$B_{i}$	0–0.4	m

**Table 3:** Weight of the parameters to be calibrated.

Parameter	Weight
$v_{i}^{o}$	0.10
$\tau_{i}$	0.05
$A_{i}$	0.74
$B_{i}$	0.11

**Table 4:** Parameter list of equipment in metro stations.

	Value	Unit
Screen doors	20	/
Platform length	83.75	m
Platform width	9.75	m
Escalator	2	/
Escalator width	1	m
Escalator speed	0.65	m/s
Stair	2	/
Stair width	1.75	m
Security checker	3	/
Turnstile	22	/
Screen doors	20	/

**Table 5:** Pedestrian parameters.

Parameter	Male	Female	Average
Time to reach downstairs (s)	14.78	15.91	15.20
Peak hourly traffic (persons)			1032
Time to pass through security check (s)	3.47	3.88	3.675
Time to travel from escalator/stairway entrance to screen door (s)	11.63	13.78	12.705
Time to travel from gate to escalator/stairs (s)	6.64	6.68	6.66
Proportion of trains waiting in the upward direction (%)			82.26
Proportion of trains waiting in the downward direction (%)			17.74
Proportion of alightings in the upward direction (%)			27.91
Proportion of alightings in the downward direction (%)			72.09

**Table 6:** Escalator/stair and directions corresponding to each area.

Area	Escalator/Stair	Downward/Upward
A	Stair 1	Upward
B	Escalator 1	Downward
C	Stair 2	Upward
D	Escalator 2	Downward

**Table 7:** Calibrated parameters of social force model.

Parameter	Value	Unit
$v_{i}^{o}$	1.37	m/s
$\tau_{i}$	0.53	s
$A_{i}$	10.25	ms^−2^
$B_{i}$	0.28	m

## Data Availability

The data that support the findings of this study are available from the funding upon reasonable request.
